# Low-Volume Brachial Plexus Block Providing Surgical Anesthesia for Distal Arm Surgery Comparing Supraclavicular, Infraclavicular, and Axillary Approach: A Randomized Observer Blind Trial

**DOI:** 10.1155/2016/7094121

**Published:** 2016-11-21

**Authors:** Mojgan Vazin, Kenneth Jensen, Danja L. Kristensen, Mathias Hjort, Katrine Tanggaard, Manoj K. Karmakar, Thomas F. Bendtsen, Jens Børglum

**Affiliations:** ^1^Department of Anesthesia and Intensive Care Medicine, Copenhagen University Hospital, Bispebjerg, Bispebjerg Bakke 23, 2400 Copenhagen NV, Denmark; ^2^Department of Anesthesia and Intensive Care Medicine, Zealand University Hospital, University of Copenhagen, Sygehusvej 10, 4000 Roskilde, Denmark; ^3^Department of Anesthesia & Intensive Care, The Chinese University of Hong Kong, Prince of Wales Hospital, Shatin, New Territories, Hong Kong; ^4^Department of Anesthesia and Intensive Care Medicine, Aarhus University Hospital, Nørrebrogade 44, 8000 Aarhus, Denmark

## Abstract

*Background*. Distal arm surgery is widely performed under regional anesthesia with brachial plexus block. The preponderance of evidence for the efficacy relies upon injection of local anesthetic in excess of 30 mL. We aimed to compare three different ultrasound-guided brachial plexus block techniques restricting the total volume to 20 mL.* Methods*. 120 patients were prospectively randomized to ultrasound-guided brachial plexus block with 20 mL ropivacaine 0.75% at either the supraclavicular, infraclavicular, or axillary level. Multiinjection technique was performed with all three approaches. Primary outcome measure was performance time.* Results*. Performance time and procedural pain were similar between groups. Needle passes and injection numbers were significantly reduced in the infraclavicular group (*P* < 0.01). Nerve visibility was significantly reduced in the axillary group (*P* = 0.01). Success-rate was significantly increased in the supraclavicular versus the axillary group (*P* < 0.025). Total anesthesia-related time was significantly reduced in the supraclavicular compared to the infraclavicular group (*P* < 0.01). Block duration was significantly increased in the infraclavicular group (*P* < 0.05). No early adverse effects occurred.* Conclusion*. Supraclavicular and infraclavicular blocks exhibited favorable characteristics compared to the axillary block. Supraclavicular brachial plexus block with the multiinjection intracluster technique exhibited significantly reduced total anesthesia-related time and higher success rate without any early adverse events.

## 1. Introduction

Ultrasound-guided (USG) brachial plexus (BP) blocks are common regional anesthesia techniques for surgical anesthesia and postoperative pain management for elbow, forearm, wrist, and hand surgery. USG BP blocks for distal arm surgery are typically administered at the supraclavicular (SC), infraclavicular (IC), or axillary (AX) level. The efficacy of these three techniques has been compared in several previous studies relative to a wide range of outcome variables [1–10]. A very recent systematic review of 25 randomized controlled trials of USG BP blockade found no differences in the rate of successful blockade with approach or with number of injections [11]. In the same review, only one randomized controlled trial was reported to have compared all three techniques in the same study [1, 11]. Tran et al. [1] found no differences between groups with respect to total anesthesia-related time, success rate, and block-related pain scores. However, Tran et al. [1] used a total of 35 mL of local anesthetic (LA) for all three USG BP blocks and reported a significantly higher incidence of Horner's syndrome. Koscielniak-Nielsen et al. [3] compared SC and IC BP blocks using a total of 30–50 mL of LA and reported a significantly higher rate of success for the IC compared to the SC BP block but a concomitant significantly higher incidence of early adverse effects with the SC BP block, that is, paraesthesia (54%), Horner's syndrome (29%), and phrenic nerve palsy (12%). Several previous studies have investigated whether single or multiple injections affected success rate and total anesthesia-related time in SC, IC, and AX BP blocks and have found no significant difference in this respect. However, the vast majority of these studies have used injection of LA in excess of 30 mL [8, 12–21].

The aim of our study was to compare several clinically important outcome parameters resulting from the administration of one of the three different USG BP block techniques, using the multiple injection technique. Importantly, we restricted the volume of LA to a total of 20 mL for all blocks. It is one of the many advantages of ultrasound in regional anesthesia that lower volumes of LA can be used, and thus reducing the risk of adverse events and LA systemic toxicity. We hypothesized* a priori* that the USG SC BP block was faster to perform compared to the IC and AX blocks, since the BP at the SC level is situated very superficially and all neural structures are easily visualized.

## 2. Methods

This randomized observer blind trial was approved by the Regional Research Ethics Committee of Copenhagen, Denmark, by Chairman Simon Francis Thomsen on 15 August 2012 (H-2-2012-055) and registered with ClinicalTrials.gov (NCT01993290). The study was conducted at Copenhagen University Hospital, Bispebjerg, Denmark.

### 2.1. Patients

Patients scheduled to undergo acute or elective surgery of the elbow, forearm, and hand in awake anesthesia were invited to participate in this study from April 2013 until April 2014. The inclusion criteria were ASA physical status classification I–III and age ≥18 years. Exclusion criteria were inability to cooperate, inability to read and understand Danish, allergy to any drugs used in the study, BMI > 35, pregnancy or nursing, peripheral neuropathy, neurological disorders, infection, coagulopathy, and a history of serious alcohol and drug dependency.

### 2.2. Design

After providing oral and written informed consent on the day of surgery, patients were randomized to one of three preoperative BP blocks. The randomization was performed using a physical method with 120 sealed opaque envelopes containing group allocation to one of three USG BP blocks. Upon sealing the envelopes prior to the randomization process, they had all been mixed and subsequently supplied with a number from 1–120 by a consultant anesthetist not affiliated with the study. We had no changes in methodology after the trial commencement.

### 2.3. Study Parameters

The primary outcome measure was performance time (seconds) from transducer placement on the skin until needle retraction. An investigator blinded to block allocation performed the time measurement. Secondary outcome measures were as follows: (i) visibility of nerve structures, 2 = good, 1 = medium, and 0 = poor, was assessed by the block administrator just prior to the block administration. (ii) Block onset-time (degree of sensory and motor block accomplished) was assessed at 10, 20, 30, and, if necessary, 40 minutes by an investigator blinded to block allocation, comparing the affected arm with the contralateral arm. The motor block was assessed by evaluating the selective movement at the level of the elbow, wrist, and fingers; that is, 2 = normal, 1 = reduced, and 0 = paralysed. Sensory block assessment was recorded on a specified chart as the degree of dermatome anesthesia using cold ethanol on skin; that is, + is reduced/abolished sensation and ÷ is normal sensation. (iii) Number of needle passes was defined as changes of the needle direction while performing the block. (iv) Total number of LA injections administered during the block procedure as registered by an observer was recorded. (v) Procedural pain, 2 = painful, 1 = acceptable, 0 = no pain, was recorded just after the block placement. (vi) Immediate adverse events relative to the block procedure were as follows: pneumothorax, accidental vascular puncture, Horner's syndrome, allergic reactions, signs of LA toxicity, recurrent laryngeal nerve, and/or phrenic nerve paralysis. (vii) Success rate was recorded (failure defined as surgical anesthesia not manifesting at 40 minutes, block supplementation after 40 minutes, conversion to general anesthesia during surgery, or the necessity to supplement with IV analgesics during the surgical procedure). (viii) Total anesthesia-related time was calculated as performance time (sec) + time to sensory block (sec). (ix) Duration of block effect was recorded; that is, patients were asked to record when they first registered sensory inputs (e.g., pain and itching). Patients received a prestamped envelope with a questionnaire and were asked to post the letter on the day following the surgery. (x) A telephone interview and a thorough search in the electronic patient file were conducted three months following the surgical procedure. Any late adverse events such as suspected nerve injury, persistent pain, and reduced motor abilities were recorded.

### 2.4. Anesthetic Procedures

Patients were randomized to the administration of an USG IC, SC, or AX BP block prior to surgery. One of three consultant anesthetists (MV, KJ, and JB) performed all block procedures and had each performed more than 1,000 block procedures without assistance or supervision. Patients were transferred to the dedicated block preparation room one hour prior to the expected surgical procedure and monitored with 3-lead electrocardiogram, pulse oximetry, and noninvasive blood pressure. A 22 G intravenous catheter was placed in the nonaffected arm.

### 2.5. Surgical Procedures

These consisted of several procedures evenly divided between the three intervention groups (Table 2).

### 2.6. Study Interventions

All patients were subjected to one of the three blocks using a short axis, in-plane technique in the supine position. All USG blocks were performed using a SonoSite EDGE ultrasound unit (SonoSite Inc., Bothell, Washington) with a linear array transducer (6–15 MHz, HFL 50). A 21-gauge, 90 mm needle (Polymedic ultrasound needle with 30 degree bevel; Temena SAS, Carrières-sur-Seine, France) was used for all blocks. Before the nerve block administration, the block area was swapped with 2% chlorhexidine/70% isopropyl alcohol. Midazolam 1-2 mg IV was offered and administered at the request of the individual patients. For all blocks, a single skin penetration and multiple injection technique was used and a total of 20 mL of ropivacaine 0.75% was administered.

**Figure 1:**
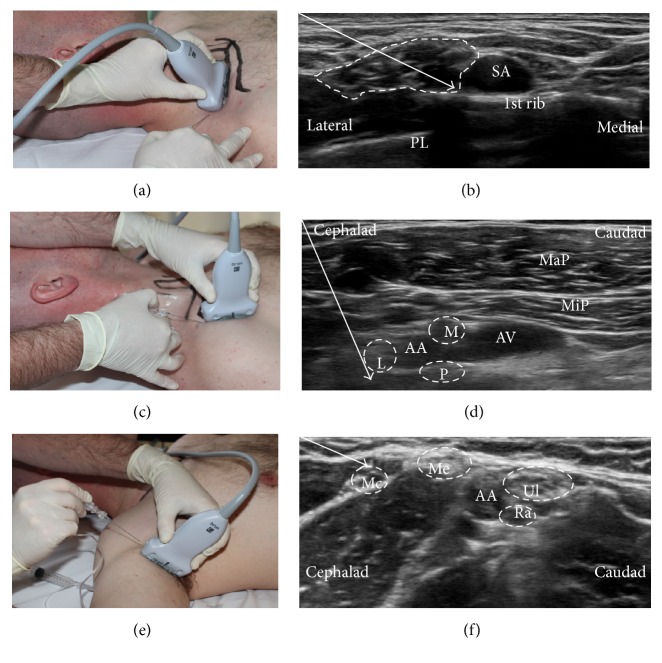
Ultrasound-guided brachial plexus blocks using a short axis, in-plane technique. Supraclavicular brachial plexus block (a) with the transducer parallel with the clavicle and with needle tip positioned subfascial and intracluster injection (b). Infraclavicular brachial plexus block (c) with the transducer in the deltopectoral groove and with needle tip position (d) close to the axillary artery (AA) at each of the cords, that is, lateral (L), medial (M), and posterior (P) cord. Axillary brachial plexus block (e) with needle tip position (f) at each of the four individual nerves, that is, musculocutaneous (Mc), median (Me), ulnar (Ul), and radial (Ra) nerve. Pl: pleura; MaP: major pectoral muscle; MiP: minor pectoral muscle; AV: axillary vein.

For the SC block (Figure 1(a)), the transducer was placed in the supraclavicular fossa parallel to the clavicle. The BP was located (Figure 1(b)). SC block sequence is as follows: (i) the needle was inserted in-plane and lateral to the transducer with a 20–25-degree angle and directed medially towards the cluster of multiple divisions of the BP contained within the hyperechoic perineural fascial sheath. This constitutes a so-called intracluster or subfascial injection of LA. The tip of the needle then penetrated the fascial sheath and was advanced gradually while injecting small boluses of LA to move the hypoechoic nerve divisions away until contact with the first rib was clearly visualized, thus using the intracluster technique described very recently [17, 22, 23]. (ii) The needle was then retracted and the trajectory subsequently changed in the same fashion with as many needle passes as needed to surround all the neural structures subfascially with LA.

For the IC block, the affected arm was abducted 90 degrees and the elbow flexed sufficiently, thereby exposing the BP (Figure 1(c)). The ultrasound transducer was positioned below the clavicle in the deltopectoral groove (Figure 1(c)). The cords of the BP were then located just deep to the minor pectoral muscle surrounding the axillary artery (Figure 1(d)). IC block sequence is follows: (i) the needle was inserted in-plane to and cephalad to the transducer with a 50–60-degree angle to the footprint of the transducer and directed towards the posterior cord at the six o'clock position deep to the artery, and, at this position, 10 mL of LA was injected to surround the posterior cord. (ii) The needle was then retracted in the same trajectory to the position just cephalad to the lateral cord, and at this position 5 mL of LA was injected to surround the lateral cord. (iii) The needle was then retracted further ending within the minor pectoral muscle, and the trajectory subsequently changed to a shallower angle (35–40 degrees) and advanced towards the position of the medial cord, and at this position the final 5 mL of LA was injected to surround the medial cord.

For the AX block, the affected arm was abducted 90 degrees sufficiently to expose the axilla with the elbow flexed. The axillary artery and veins were identified with the surrounding nerves and muscles (Figure 1(e)). The needle was inserted in-plane to and cranial to the transducer with a 15–20-degree angle to the footprint of the transducer and directed caudad (Figure 1(f)). AX block sequence: (i) the needle tip was first advanced to the musculocutaneous nerve while visualizing the injectate of 5 mL of LA surrounding the nerve. (ii) The needle was then retracted and the trajectory subsequently changed and advanced towards the axillary artery where the needle tip was subsequently directed towards the median, the ulnar, and finally the radial nerve, visualizing an injectate of 5 mL of LA surrounding each nerve (Figure 1(f)).

### 2.7. Statistical Analysis

In a pilot study performed on 30 patients where all three USG BP block techniques were equally distributed, we recorded a performance time between 120 and 480 seconds with a mean of 260 seconds. We considered a Minimal Relevant Difference (MIREDIF) of 15% clinical relevant, which is equal to selecting 40 seconds as a clinically relevant difference (40/260 = 15.38%). This correlated fairly well with the assumptions from a previous study [1]. Assuming a 5% significance level, a power of 80%, and a standard deviation of (260 *∗* 0.95/4 = 62) 62 seconds, we calculated a total sample size of 38 patients in each group. Thus, allowing for a 5% dropout rate, a total of 120 patients were included in the trial. Normality of all continuous data was first assessed with the Anderson-Darling test. If this test did not consistently support a Gaussian distribution for the continuous variables, results were presented by median [IQR]; regardless of the distribution, continuous data was analyzed using a one-way ANOVA, with post hoc testing using Tukey's HSD test for multiple comparisons. Categorical variables were presented as numbers (and %) and analyzed using a 2 × 2 or 2 × 3 contingency table with a two-tailed Fisher's exact test. *P* < 0.05 was considered statistically significant for all comparisons, and no additional adjustment for multiple comparisons was used for categorical variables. Sensory and motor results were presented graphically.

## 3. Results

**Figure 2:**
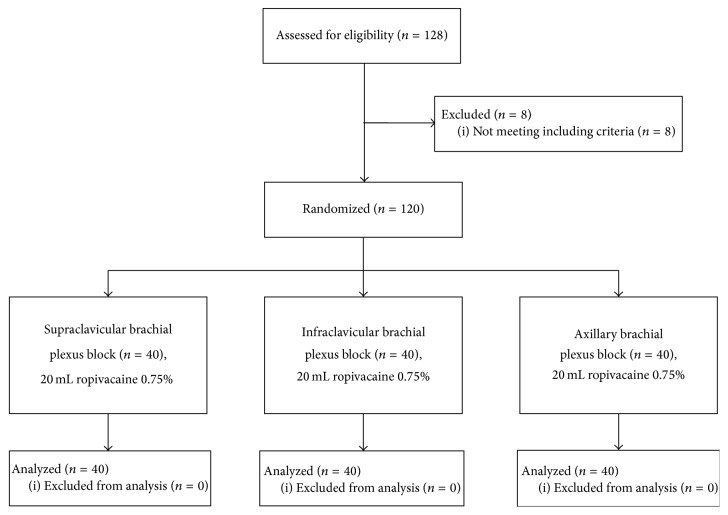
CONSORT diagram.

**Figure 3:**
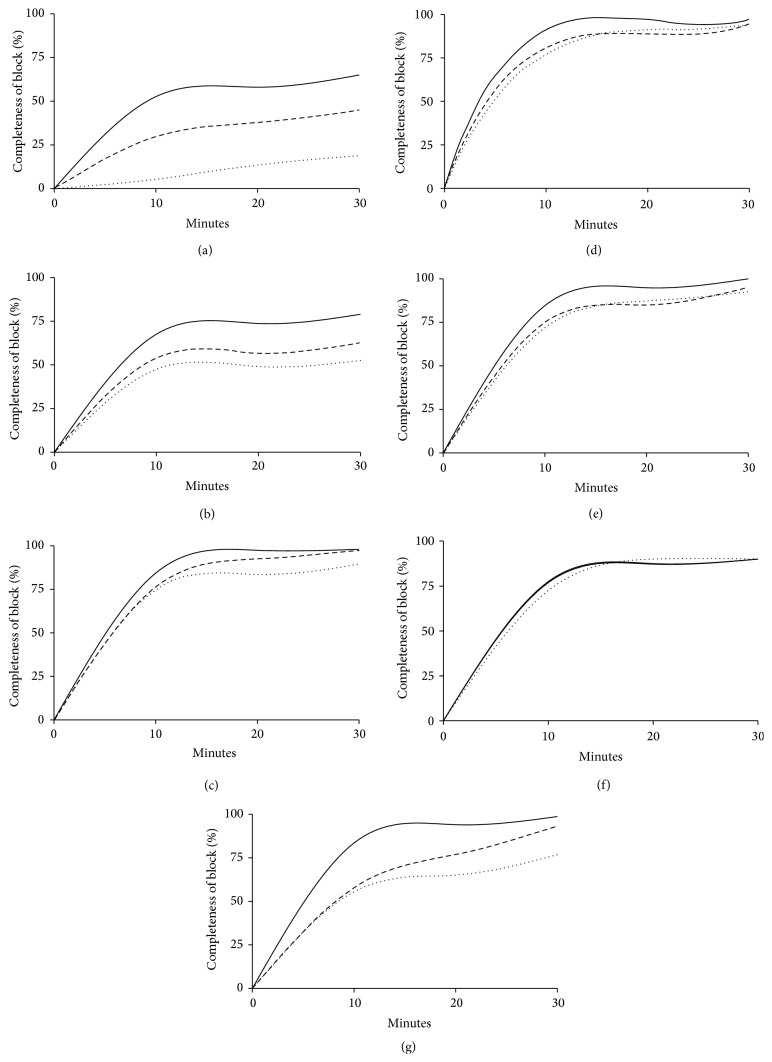
Completeness of sensory block of individual nerves 0–30 minutes after block administration. Completeness of sensory block over time for each major nerve of the upper extremity. (a) Axillary nerve, (b) intercostobrachial and medial brachial cutaneous nerves, (c) medial antebrachial cutaneous nerve, (d) musculocutaneous nerve, (e) median nerve, (f) ulnar nerve, and (g) radial nerve. Supraclavicular block: straight line; infraclavicular block: dashed line; axillary block: dotted line.

**Figure 4:**
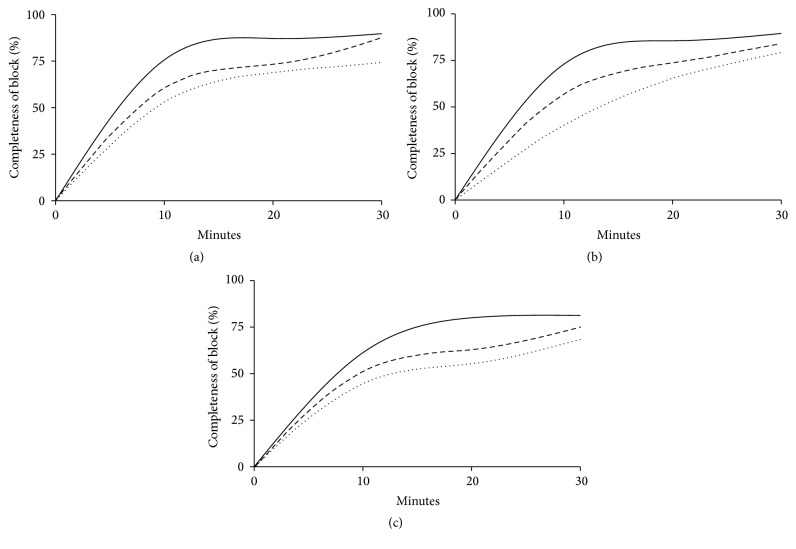
Completeness of motor block in elbow, wrist, and fingers 0–30 minutes after block administration. Completeness of motor block over time for each major joint. (a) Elbow, (b) wrist, and (c) fingers. Supraclavicular block: straight line; infraclavicular block: dashed line; axillary block: dotted line.

One hundred and twenty-eight patients scheduled to distal arm surgery in awake anesthesia were screened for eligibility. Six patients were excluded due to inability to read and understand Danish, and two patients were excluded due to a history of serious alcohol and drug dependency. One hundred and twenty patients were eventually included and were equally randomized into the three groups according to block allocation. Patient flow throughout the study is presented in Figure 2. Patient demographics are presented in Table 1. The various surgical procedures are presented in Table 2. Main results are summarized in Tables 3 and 4. Completeness of sensory and motor block over time for each block approach is graphically displayed in Figures 3 and 4. All continuous distributions, except duration of analgesia, were estimated to be non-Gaussian.

Performance time and procedural pain were similar in all groups. The IC BP block technique was performed with significantly fewer needle passes as well as fewer injections of LA compared to the other two techniques (*P* < 0.01). The overall visibility of the neural target structures in the ultrasound image was significantly reduced for the AX BP block compared to the other two approaches (*P* = 0.01); that is, visibility and completeness of sensory block were reduced for the radial nerve in particular. Block onset-time and total anesthesia-related time were significantly faster for the SC BP approach compared to the IC BP approach; *P* < 0.01 and *P* < 0.05, respectively. Success rate of surgical anesthesia was also significantly higher for the SC BP approach compared to the AX BP approach (*P* < 0.025). The IC BP block had significantly longer duration of analgesic effect compared to the two other approaches (*P* < 0.05). No early adverse effects were recorded in any of the groups during and after administration of the blocks. A total of 21 (17%) late adverse events such as suspected nerve injury, persistent pain, or reduced motor function were recorded. However, only 10 (8%) of the cases with suspected nerve injury could possibly be attributed to the regional anesthesia procedures, and the severity of the injuries was assessed by the surgeons to be too insignificant for referral to the neurophysiology examination in all cases.

## 4. Discussion

### 4.1. Block Characteristics

The primary outcome of performance time was not statistically different between groups, even though the ultrasonographic visibility of the neural structures was significantly reduced with the AX BP approach compared to the IC and SC approaches; that is, the latter two were comparable in this regard (Table 3). Previously published data about performance time vary considerably between studies [1–10]. The visibility scores demonstrated that the radial nerve in the AX BP block was the neural structure most difficult to visualize. Importantly, a previous publication also found success rate for USG AX BP block similar to our results [24]. Chan et al. [24] showed that it was particularly evident that the failure was due to a lack of achieving radial nerve anesthesia with the USG AX BP approach. The neural structures of the BP at the SC level are seen as the multiple divisions within the hyperechoic perineural fascia, and it can be difficult to anatomically divide the plexus into separate units at this level. For the IC BP block, the medial cord of the BP appeared to be slightly less visible than the other cords (Table 3).

No statistically significant difference in procedural pain was detected between the three approaches. We compared three single-needle-skin-penetration and multiple injection techniques using a nontraumatic needle with a 30-degree bevel, using no skin local anesthesia and offering only 1-2 mg midazolam as a sedative. None of our patients had IV opioids during the block procedure or during surgery. Previous studies likewise suggest that procedural pain is similar for various approaches to block the BP [1, 4, 6, 8]. Our results imply that it is not so much the depth of the neural structures, the number of needle passes, or the number of injections administered that influence the patient perspective. The three consultant anesthetists who performed all the blocks are all experts of USG peripheral nerve blocks, which can limit the external validity of the study [4, 5, 7, 9].

### 4.2. Postblock Characteristics

The SC BP block had a significantly faster block onset-time compared to the IC BP block (Table 4). We speculate that our findings may be related to the fact that, with the SC BP approach, the LA is deposited as an intracluster injection at several positions after having penetrated the perineural fascial sheath surrounding the multiple divisions of the BP [17, 22, 23]. Penetration of the perineural fascial sheath at the SC level was first described by Kapral et al. in 1994, who reported just one needle pass and one injection [7]. Eight studies have reported a penetration of the perineural fascial sheath at the SC level [7, 17, 18, 23, 25–27], but only two studies have employed more than two needle passes within the perineural fascial sheath at this level [17, 18].

Other studies have compared BP blocks at the AX, IC, and SC levels [1, 28]. None of these consistently used multiple injection technique at all levels, with a total volume of just 20 mL of LA. Koscielniak-Nielsen et al. [3] utilized another USG technique when comparing block efficacy of the SC BP and IC BP block approaches. Half of the injected LA volume was deposited superficially to the plexus during the SC BP block, whereas the other half was injected perineurally. The authors found significantly faster onset, better surgical effectiveness, and fewer physiological effects favoring the IC BP block compared to the SC BP block. However, a total volume of 30–50 mL of LA was used, which may explain the significantly higher incidence of early adverse effects with the SC BP block approach [1, 3]. Further, our own results showed that the SC BP block resulted in a significantly higher success rate of surgical anesthesia compared to the AX BP block, while the IC BP and SC BP approaches demonstrated equal success rates (Table 4).

### 4.3. Immediate and Late Adverse Events

We recorded no immediate adverse events during and after block administration. The observation period for these contingent immediate and early adverse events spanned the time in the block preparation area, in the operating theatre, and in recovery. Previous studies have reported a significantly higher number of early adverse effects associated with the SC BP approach [1, 3, 10], which has been a pivotal argument for opting out the SC BP technique in many surgical centers. Several dose-finding studies have been conducted using less than 20 mL of LA, but only one has compared various techniques for BP blockade [5] and only two studies reported on physiological adverse effects [25, 28]. To the best of our knowledge, the present study is the first randomized controlled trial showing that the SC block approach to BP blockade, with a single penetration and multiple injection intracluster technique, is superior with regard to clinically relevant efficacy measures and frequency of early adverse events compared to other low-volume BP block techniques.

### 4.4. Limitations

Firstly, as with other procedure-related studies, blinding the operator to group allocation was not possible. To minimize the risk of performance bias, the three anesthesiologists performing the blocks adhered to a strict protocol when administering the LA. Despite this measure, performance bias cannot be ruled out. Secondly, with performance time as the primary outcome measure and a MIREDIF of 40 seconds, it can be discussed whether this difference is actually clinically relevant. However, shorter time for block placement was thought to translate into lesser degree of procedural pain and patient discomfort. Thirdly, we have included one hundred and twenty patients in this study undergoing different surgical procedures (Table 2), and some of these procedures did not always affect areas innervated directly by all the nerves from the BP. Thus, a complete BP block could possibly be perceived as excessive for some of these surgical procedures. However, we took into account that several surgeons wanted arm tourniquets for most surgical procedures, disregarding the innervation of the surgical field. Fourthly, interrater reliability between the involved data collectors was not assessed in our study.

## Figures and Tables

**Table 1:** Baseline demographics.

	AX	IC	SC
Height [cm]	168 [13]	175 [16]	172.5 [13]
Weight [kg]	67 [20]	72 [20]	75 [24]
BMI [kg/m^2^]	23.9 [5.3]	22.9 [4.2]	25.1 [8.8]
Age [years]	60 [35]	52 [38]	59 [26]
ASA I-II/III-IV [*n*/*n*]	19/21	21/19	13/27

Values are reported as median [IQR] or numbers [*n*/*n*]. AX, axillary; IC, lateral infraclavicular; SC, supraclavicular; BMI, body mass index; ASA, American Society of Anesthesiology physical classification. Anderson-Darling normality test performed on all continuous data, suggesting non-Gaussian distributions.

**Table 2:** Surgical procedures.

	AX	IC	SC
Fracture, forearm [*n*]	7	7	6
Fracture, wrist [*n*]	18	14	10
Fracture, digits [*n*]	6	12	9
Soft tissue, forearm [*n*]	4	2	4
Soft tissue, wrist [*n*]	5	0	7
Soft tissue, digits [*n*]	0	5	4

Values are reported as numbers [*n*]. AX, axillary; IC, lateral infraclavicular; SC, supraclavicular. No statistical tests performed.

**Table 3:** Block characteristics.

	AX	IC	SC	ANOVA	Tukey's HSD or Fisher's exact test
Performance time [seconds]^(1)^	184 [86]	179 [83]	210 [57]	*P* = 0.307	NS
Needle passes [*n*]^(2)^	6.0 [3.0]	4.0 [2.3]	6.0 [4.0]	*P* = 0.000	AX > IC, *P* < 0.01; SC > IC, *P* < 0.01
Injections [*n*]^(3)^	9.0 [3.3]	6.0 [3.0]	9.0 [3.0]	*P* = 0.000	AX > IC, *P* < 0.01; SC > IC, *P* < 0.01
Overall visibility [good/medium/poor]^(4)^	4/8/22	17/8/15	20/14/6	n/a	AX < IC, *P* = 0.010; AX < SC, *P* < 0.0001
Visibility of substructures [good/medium/poor]	Musc.cut. 28/3/3, median 20/13/1, ulnar 15/12/7, radial 6/12/16	Lateral cords 28/8/4, medial cords 21/10/9, posterior cords 27/6/7	n/a	n/a	n/a
Procedural pain [no pain/acceptable/painful]^(5)^	20/18/2	20/16/4	25/12/3	n/a	NS

*P*-values for the Anderson-Darling normality test results: (1) performance time: AX, *P* < 0.0005; SC, *P* = 0.497; IC, *P* = 0.588; (2) needle passes: AX, *P* = 0.058; SC, *P* = 0.056; IC, *P* < 0.0005; (3) aliquots: AX, *P* = 0.458, SC, *P* = 0.117; IC, *P* = 0.020. Continuous variables are reported as median [interquartile range], and comparisons are analyzed by one-way ANOVA and subsequent Tukey HSD test. For categorical variables, comparisons are analyzed by two-tailed Fisher's test. Additional Fisher's tests: (4) visibility: IC = SC, *P* = 0.060; (5) procedural pain: AX = IC, *P* = 0.705; AX = SC, *P* = 0.377; IC = SC, *P* = 0.594. AX, axillary; IC, infraclavicular; SC, supraclavicular; *n*, numbers; n/a, not applicable; NS, not significant (*P* > 0.05).

**Table 4:** Postblock characteristics and adverse effects.

	AX	IC	SC	ANOVA	Tukey's HSD or Fisher's exact test
Time to sensory block [minutes]^(1)^	30 [15]	30 [5]	20 [9]	0.007	IC > SC, *P* < 0.01
Total anesthesia-related time [seconds]	1957 [646]	2043 [793]	1598 [523]	0.016	IC > SC, *P* < 0.05
Duration of analgesia [hours]^(2)^	11:15 [5:51]	13:42 [7:55]	11:27 [3:22]	0.004	IC > AX, *P* < 0.05; IC > SC, *P* < 0.05
Success rate [*n*/*n*]^(3)^	30/40 [75%]	36/40 [90%]	38/40 [95%]	n/a	SC > AX, *P* = 0.025
Early adverse effects [*n*]	0 [0%]	0 [0%]	0 [0%]	n/a	NS
Late dysesthesia potentially linked to nerve blocks [*n*]^(4)^	4 [10%]	5 [13%]	1 [3%]	n/a	NS
Late paralysis [*n*]	1 [3%]	0 [0%]	0 [0%]	n/a	NS

*P* values for the Anderson-Darling normality test are as follows: (1) time to sensory block: AX, *P* = 0.153; IC, *P* < 0.0005; SC, *P* < 0.0005; (2) duration of analgesia: AX, *P* = 0.569; IC, *P* = 0.367; SC, *P* = 0.877. Continuous variables are reported as median [interquartile range], and comparisons are analyzed by one-way ANOVA and subsequent Tukey's HSD test. For categorical variables, comparisons are analyzed by two-tailed Fisher's test. Additional Fisher's tests are as follows: (3) success rate: AX = IC, *P* = 0.140; IC = SC, *P* = 0.675; (4) late dysesthesia: AX = IC, *P* = 1.000; AX = SC, *P* = 0.359; IC = SC, *P* = 0.201. AX, axillary; IC, infraclavicular; SC, supraclavicular; *n*, numbers; n/a, not applicable; NS, not significant (*P* > 0.05).
